# 基于RNA-based NGS检测非小细胞肺癌融合基因临床实践中国专家共识

**DOI:** 10.3779/j.issn.1009-3419.2023.102.43

**Published:** 2023-11-20

**Authors:** 中国初级卫生保健基金会肿瘤精准诊疗专业委员会, 中国抗癌协会肿瘤精准治疗专业委员会, 中国抗癌协会肿瘤病理专委会肺癌学组

**Keywords:** 非小细胞肺癌, 融合基因, RNA-based NGS, 靶向治疗, 专家共识, Non-small cell lung cancer, Fusion genes, RNA-based NGS, Targeted therapy, Expert consensus

## Abstract

基于RNA水平的二代测序（RNA-based next-generation sequencing, RNA-based NGS）技术已被非小细胞肺癌（non-small cell lung cancer, NSCLC）临床实践指南和专家共识推荐为融合基因的检测方法之一。NSCLC可用药靶点主要包括基因突变和融合，用于评估靶向治疗可行性的基因突变和融合基因检测均不可或缺。目前，基于DNA水平的NGS（DNA-based NGS）结合RNA-based NGS一次性同步检测基因突变和融合的技术已部分应用于临床实践。然而，RNA-based NGS检测融合基因的应用时机、应用场景和质控方面在我国仍缺乏规范和标准。本共识将进一步明确RNA-based NGS在融合基因检测中的应用时机、应用场景和质控，并给予指导性建议，推动RNA-based NGS在NSCLC临床诊疗中的应用，使患者能够最大程度地从融合基因检测中获益。

## Abstract (en)

RNA-based next-generation sequencing (NGS) has been recommended as a method for detecting fusion genes in non-small cell lung cancer (NSCLC) according to clinical practice guidelines and expert consensus. The primary targetable alterations in NSCLC consist of gene mutations and fusions, making the detection of gene mutations and fusions indispensable for assessing the feasibility of targeted therapies. Currently, the integration of DNA-based NGS and RNA-based NGS allows for simultaneous detection of gene mutations and fusions and has been partially implemented in clinical practice. However, standardized guidelines and criteria for the significance, application scenarios, and quality control of RNA-based NGS in fusion gene detection are still lacking in China. This consensus aims to provide further clarity on the practical significance, application scenarios, and quality control measures of RNA-based NGS in fusion gene detection. Additionally, it offers guiding recommendations to facilitate the clinical implementation of RNA-based NGS in the diagnosis and treatment of NSCLC, ultimately maximizing the benefits for patients from fusion gene detection.

肺癌在我国发病率和死亡率均位居第一^[1]^。近十年，靶向治疗极大改善了非小细胞肺癌（non-small cell lung cancer, NSCLC）患者的预后。除了表皮生长因子受体（epidermal growth factor receptor, EGFR）、V-raf鼠肉瘤病毒癌基因同源体B（vrafmurine sarcoma viral oncegene homolog B, BRAF）等基因突变类型，在NSCLC中针对间变性淋巴瘤激酶（anaplastic lymphoma kinase, ALK）、c-ros肉瘤致癌因子-受体酪氨酸激酶（ROS proto-oncogene 1, receptor tyrosine kinase, ROS1）、转染时重排（rearranged during transfection, RET）等融合基因的靶向药物也陆续在国内外获批上市并应用于临床，为患者带来了更多的治疗机会，显著改善了携带融合基因变异患者的生存质量和预后。因此，融合基因检测越来越受到关注。脱氧核糖核酸（deoxyribonucleic acid, DNA）、核糖核酸（ribonucleic acid, RNA）和蛋白表达检测是判断基因融合的主要检测方法。目前，融合基因的金标准检测方法为基于DNA水平的荧光原位杂交（fluorescence in situ hybridization, FISH）检测，然而由于NSCLC有众多的驱动基因和多样的变异形式，因此具有高通量优势的第二代测序技术（next-generation sequencing, NGS）也广泛应用于NSCLC融合基因检测。相比于基于DNA水平的二代测序（DNA-based NGS）检测，美国国立综合癌症网络（National Comprehensive Cancer Network, NCCN）公布的NSCLC临床实践指南（2023年第5版）已提出基于RNA水平的NGS（RNA-based NGS）检测可能会提高ROS1、RET、神经营养因子受体络氨酸激酶（neurotrophin receptor kinase, NTRK）基因融合和间质-上皮细胞转化因子（mesenchymal-epithelial transition factor, MET）基因14号外显子跳跃突变（MET 14跳突）的检出率。该指南同时指出，对于在广泛组合的检测中未发现驱动基因的患者（尤其是非吸烟者）考虑采用RNA-based NGS检测，以最大程度地发现融合基因突变。此外，多项前瞻性研究^[2⇓-4]^均应用了RNA-based NGS技术作为融合基因的检测方法。例如，PROFILE 1001 I期研究和VISION II期研究均应用了RNA-based NGS检测NSCLC中ROS1基因融合和MET 14跳突。关于ALK、RET、神经调节蛋白1（neuregulin-1, NRG1）和NTRK融合基因的多项回顾性研究也应用了RNA-based NGS检测^[5⇓⇓⇓⇓⇓-11]^。目前NSCLC可用药的变异形式主要包括基因突变和基因融合，RNA-based NGS检测基因融合应整合到DNA-based NGS检测基因突变中，以提高基因融合的检出率。目前，一次性取样并通过DNA-based NGS和RNA-based NGS同时检测的技术已研发成功并开始应用于临床实践。虽然在近几年内RNA-based NGS已被报道可用于NSCLC的融合基因检测，但我国RNA-based NGS检测融合基因的应用时机、应用场景和质控尚无行业技术标准、相关规范和共识。因此，本共识对RNA-based NGS检测融合基因的应用时机、应用场景和质控进行了详尽阐述，同时提出RNA-based NGS质控和应用尚存问题和研究建议，帮助临床明确应用RNA-based NGS检测融合基因的实践意义，以期指导临床进行规范准确的RNA-based NGS检测，使携带融合基因变异的NSCLC患者最大化获益。

## 1 NSCLC的主要融合基因类型

基因融合是通过染色体反转（chromosomal inversion）、串联复制（tandem duplication）、缺失（deletion）或易位（translocation），合并不同的、独立的基因或基因片段的过程，即由于某种机制（如染色体结构重排引起基因组变异）造成两个或多个不同基因的部分序列或全部序列（编码区）首尾相连，置于同一套调控序列（包括启动子、增强子、核糖体结合序列、终止子等）控制之下，构成一个新的嵌合基因^[12,13]^。基因融合与各种疾病，尤其与癌症的发生发展密切相关^[13]^。融合基因常见于血液肿瘤和实体瘤中，实体瘤的呼吸系统肿瘤和乳腺肿瘤中融合基因出现数量相对更多^[14]^。在NSCLC中，常见的驱动融合基因包括ALK、ROS1、RET、NTRK和NRG1等^[15,16]^。此外，有研究^[17,18]^证实EGFR、BRAF、MET等常见的点突变驱动基因也会发生基因融合变异。值得注意的是，MET 14跳突从RNA水平上表现为13号外显子和15号外显子发生融合，导致MET蛋白泛素化降解受抑制^[19]^。因此，在本共识中将MET 14跳突也纳入融合基因讨论范围。总体而言，10%-15%的NSCLC患者携带基因融合变异^[20]^。

## 2 融合基因常用检测方法

目前临床常用融合基因检测方法包括免疫组织化学（immunohistochemistry, IHC）、FISH、逆转录聚合酶链反应（reverse transcription-polymerase chain reaction, RT-PCR）和NGS^[21]^，近年来，单细胞基因融合检测技术和nCounter技术也出现在融合基因检测相关研究中^[22,23]^。

### 2.1 IHC、FISH和RT-PCR

IHC通过特异性单克隆抗体检测融合蛋白表达情况，具有耗时短、成本低、自动化等优点，可作为一些融合基因的伴随诊断（如：ALK融合，D5F3克隆，Ventana平台检测）或筛查手段（如：ROS1融合和NTRK融合）。但在缺乏特异性强的检测抗体时，IHC的假阳性率和假阴性率均较高（如RET融合）^[24,25]^。针对筛查目的进行的IHC检测阳性结果，需进一步使用其他分子检测手段进行验证。FISH通过荧光标记的DNA探针来鉴别目标核苷酸序列，可检测拷贝数扩增和染色体结构改变（例如基因重排），其结果具有一定的可靠性及稳定性，对于驱动融合伴侣未明确的融合形式也可检出，目前是检测融合基因的重要手段，如ALK断裂探针（break-apart）被认为是ALK融合检测的金标准。然而，FISH技术也存在一定的局限性。例如，某些罕见或未知的断裂与融合位点间距可能小于FISH可判读的最小阈值，从而导致假阴性的产生。此外，检测结果为复杂的信号模式的判读对于诊断医师的经验依赖性很高^[23]^。RT-PCR检测基因融合时可明确融合伴侣，具有较高的灵敏度和特异性，然而RT-PCR仅能检出已知融合形式^[14,26]^。因此，以上融合基因检测方法既有其各自优势，也存在一定的局限性。

### 2.2 NGS

NSCLC融合基因数目较多，因此可以同时检测多个基因融合变异的NGS也是一种被普遍应用的检测手段。同时，当FISH和IHC判读融合基因结果不一致时，NGS是一种有效的替代方案。例如，Frampton等^[27]^及Smuk等^[28]^报道了NGS方法作为克服FISH检测的挑战性的有效手段。通过FISH检测的59例ALK基因融合样本中出现了5%的非典型减弱孤立3’信号。为了明确这类患者从靶向药物潜在获益的可能性，对这些样本同时进行了NGS检测。结果发现，NGS证实了ALK融合的存在。此外，NGS可检出已知和未知的融合基因类型，弥补常规检测方法通常无法检测或无法明确融合伴侣基因的缺陷。NGS不仅可以识别特定的靶向变异，还可识别共存的其他基因变异，如TP53突变可能会减弱ALK融合的NSCLC的靶向治疗疗效^[27,29]^。

NGS检测对象为DNA和RNA。根据中心法则，以DNA为模板通过转录和剪接加工产生成熟的RNA^[30]^。在DNA水平上，融合断点位置通常发生在较长的内含子区域，且融合断点在不同患者中具有多样性，采用NGS的方法设计探针去抓取断裂点是一种可行的检测方法。然而，从DNA水平检测融合基因不可避免存在如下局限性和挑战：（1）融合基因检测要全面覆盖非常冗长且含有大量重复序列的内含子区域，才能准确地找到融合断裂点；（2）内含子区域GC含量变化不利于探针均匀有效地捕获目标区域片段；（3）不同基因的内含子含有非常相似的重复序列，这一特征不利于序列准确对比，影响检测准确性；（4）复杂的转录或转录后的剪接加工过程，可能会影响融合基因检出^[31]^。RNA-based NGS则有望弥补这一不足，用于RNA-based NGS检测的探针仅覆盖外显子，探针设计难度较DNA更低，可检出转录水平剪切形成的基因融合变异。对于DNA-based NGS未检出融合基因的情况下，RNA-based NGS能够提高融合基因的检出率^[32,33]^。RNA-based NGS检测的样本多是基于福尔马林固定的石蜡包埋（formalin-fixed paraffin-embedded, FFPE）组织样本，长时间保存样本带来的RNA降解成为临床目前关心的问题之一^[34]^。NGS文库构建流程包括扩增子法和杂交捕获法等。与杂交捕获法相比，扩增子法对核酸模板的投入量要求更低，建库时间更短，从一定程度上可减少因RNA降解导致的对检测结果的影响^[35⇓-37]^。

## 3 融合基因检测适用的样本类型

NSCLC肿瘤组织检测（包括细胞蜡块）和液体活检是融合基因检测的两种样本类型。肿瘤组织检测样本包括穿刺组织、手术组织、气管镜活检组织、超声支气管镜（endobronchial ultrasound, EBUS）活检组织、支气管刷检组织、胸腔积液沉渣、腹腔积液沉渣、脑脊液沉渣和肺泡灌洗液沉渣等。液体活检样本包括外周血上清、胸腔积液上清、腹腔积液上清、脑脊液上清和肺泡灌洗液上清等。FISH、IHC这类传统检测方法采用肿瘤组织检测，NGS采用肿瘤组织检测和液体活检检测。目前，大多数RNA-based NGS检测融合基因的研究采用组织检测，仅有少数RNA-based NGS检测融合基因的研究采用液体活检进行检测^[32]^。有学者^[38]^认为基于循环肿瘤DNA（circulating tumor DNA, ctDNA）的NGS融合基因检测敏感性低于肿瘤组织检测。因此，NSCLC融合基因检测，首选肺癌肿瘤组织，ctDNA检测可作为组织样本不可及情况下的替代选择，但目前还缺乏相应的检测标准和规范^[39]^。

## 4 基于RNA-based NGS检测融合基因的专家共识

本共识基于文献数据并结合我国国情与分子检测需求，共总结6条检测相关意见要点，参与共识的临床病理专家通过共识线上会议或邮件形式对每条共识进行“非常同意”“基本同意”或“不同意”的投票。超过2/3以上专家组投票，计为有效投票，最终形成推荐意见和推荐等级。本共识分为“强烈推荐”“推荐”和“未达成共识”。共识专家组投“非常同意”的票数超过2/3的意见为“强烈推荐”，专家组投“非常同意”+“基本同意”的票数超过2/3的意见为“推荐”，否则不达成共识。投票参考推荐分级的评估、制定和评价（Grade of Recommendations Assessment, Development and Evaluation, GRADE）方法。

**共识一：相比DNA-based NGS，RNA-based NGS不受内含子影响，可提升融合基因的检出率。建议有条件的医疗机构对NSCLC样本进行一次性同步RNA-based NGS与DNA-based NGS的驱动基因变异（融合/突变）检测。【强烈推荐】**

在DNA-based NGS未检出融合基因变异的NSCLC患者中，RNA-based NGS可能检出更多融合基因变异。一项真实世界肺腺癌大队列研究^[40]^发现，在DNA-based NGS检测为驱动基因变异阴性的232例患者中，经RNA-based NGS可检测到更多融合基因变异（14.2%, 33/232），其中10例患者接受对应靶向治疗，8例患者实现临床获益。这一现象在Beaubier等^[41]^及Zacharias等^[42]^公布的研究中同样存在。在中国研究者公布的数据^[43]^中，140例基于DNA-based NGS检测的驱动基因阴性患者通过RNA-based NGS多检出了14例（10%）携带可用药融合变异。

相比DNA水平，RNA水平上融合基因表现为前后两个基因外显子之间的衔接，不受内含子的影响，融合点相对固定，这一特征为精准设计探针或引物提供了先天优势，也是RNA-based NGS比DNA-based NGS检测融合基因更具优势的原因之一。Benayed等^[40]^认为即使在大型杂交捕获面板中，由于内含子的长度和目标区域内的盲点，并非所有的融合均能被识别。Seo等^[44]^发现融合基因的RNA表达量都显著上调，其原因在于融合伴侣在自身发生转录的同时，其下游激酶区域同时发生了转录，使得激酶区域转录被激活产生了大量的mRNA，因此通过RNA检测可更容易识别到基因融合。由此可见，通过RNA-based NGS检测融合基因无需考虑内含子影响，且有概率更准确地检出融合基因。

随着NGS技术的不断进步，越来越多的罕见融合变异形式被发现^[45⇓-47]^。然而，罕见融合变异的功能及临床意义仍然存在一定争议。RNA-based NGS可验证DNA-based NGS检测的罕见融合变异形式。例如，Ding等^[48]^的研究中发现了1例经DNA-based NGS检测的患者携带ALK基因罕见融合变异形式（LANCL1-ALK, L7:A20），经RNA-based NGS验证后确定为类棘皮细胞微管相关蛋白4-ALK（echinoderm microtubule-associated protein-like 4-ALK, EML4-ALK）（E13:A20）转录本。另一项研究^[49]^同样发现了相似现象，研究者采用DNA-based NGS鉴定了14例ALK基因复杂融合形式的NSCLC患者，对保留足够标本的13例患者加测了RNA-based NGS检测，发现融合基因和断点位置在DNA和RNA测序之间存在明显差异，所有受检者实际上都表达了经典的EML4-ALK融合转录本。此外，也有案例报道^[50]^提示对于DNA水平发现罕见或复杂融合形式的ALK融合患者，需尽早加测RNA-based NGS以验证其功能。该现象在RET基因融合变异中也有相关报道。例如：一项回顾性研究^[8]^对44例携带非经典RET融合（除KIF5B-RET和CCDC6-RET之外的RET融合都被归类为非经典RET融合）的配对DNA-based NGS和RNA-based NGS测序数据进行比较分析，结果发现44例样本中有41例在RNA水平上检出了RET融合（93.2%）。其中，68%（28/41）为经典的KIF5B-RET融合，12%（5/41）为经典的CCDC6-RET融合，20%（8/41）为其他非经典融合，这表明在DNA层面检出的大部分非经典RET重排在RNA层面实质为经典RET融合（80.5%, 33/41）。在另一项研究^[7]^中，12例经DNA-based NGS检测出RET非经典融合的患者中均检测到了RET融合转录，但仅有5例患者的融合伴侣和外显子断裂点结果与RNA-based NGS结果一致；在另外20例DNA-based NGS检测出RET非经典融合的患者中，通过RNA-based NGS均没有检测到RET融合转录。因此，对于ALK和RET融合基因的罕见融合变异形式，可通过RNA-based NGS验证其融合功能。

NSCLC的靶向用药检测主要包括基因点突变、插入缺失突变和基因融合。临床实践中，利用基于DNA的检测技术（NGS或PCR）完成一次性检测较为普遍，而实际基于RNA的检测技术对融合基因检出更具优势。目前在技术层面上，使用RNA-based NGS和DNA-based NGS在一个面板中进行检测已可实现。具体而言，先对一份肿瘤组织样本同时进行RNA和DNA提取，随后将RNA反转录为cDNA，再以一定比例与DNA样本混合，加入定制引物后形成混合体系。通过扩增子建库法将混合模板进行多重PCR扩增，文库产物上机测序后即可同时进行点突变、插入缺失和融合突变的分析。 使用上述方法，DNA和RNA一次性同时检测并不对样本数量和检测成本有更高要求，目前该技术已应用于临床^[51,52]^。考虑到晚期肺癌患者肿瘤组织获取有限、治疗的及时性以及检测的经济性，建议有条件的医疗机构对NSCLC组织的一份样本一次性利用DNA-based NGS检测点突变或插入缺失突变，同步利用RNA-based NGS检测基因融合变异。

**共识二： 目前RNA-based NGS可用于检测ALK、RET、ROS1、NTRK、NRG1和MET等驱动基因融合。【强烈推荐】**

NSCLC国内外诊疗指南一致推荐检测的融合基因包括ALK、ROS1、RET和NTRK。因此，NSCLC中RNA-based NGS检测基因融合应至少涵盖上述基因。一些针对新兴靶点NRG1融合的抗体药物Zenocutuzumab也在开展临床试验，目前已在携带该靶点的胰腺癌和NSCLC患者中展现出来良好的疗效^[53⇓-55]^。对于罕见融合靶点（如EGFR、MET、FGFR和BARF），有病例报道发现NSCLC患者可从相应靶向药物中获益，一些相关临床研究^[56⇓⇓⇓⇓⇓⇓⇓⇓-65]^也正在开展。此外，MET 14跳突是由于RNA剪接异常而造成MET基因14号外显子丢失，在DNA层面的突变具有区域复杂多样性，存在功能解读困难。内含子区域突变和在转录剪接水平发生的外显子融合可能导致DNA-based NGS出现MET 14跳突的漏检现象^[66⇓-68]^。NCCN指南中也明确提出RNA-based NGS可提高MET 14跳突的检出率。因此在条件允许时，可考虑采用同时涵盖ALK、RET、ROS1、NTRK、NRG1融合和MET 14跳突的RNA-based NGS产品进行多靶点联合共检。

**共识三：由RNA-based NGS检测的融合基因可指导NSCLC融合变异相关的靶向治疗。【强烈推荐】**

由RNA-based NGS检测的融合基因可以指导融合基因变异相关的靶向治疗。一项发表在Lung Cancer上的研究^[69]^结果显示，由RNA-based NGS检测ALK融合基因的患者接受克唑替尼治疗的中位无进展生存期（progression-free survival, PFS）显著优于ALK融合基因未检出患者（182天 vs 20天，P<0.0001），中位持续治疗时间也显著长于ALK融合基因未检出患者（230天 vs 20天，P<0.0001）。同时，研究发现通过克唑替尼治疗的EML4-ALK V1/V2患者（n=6）比V3a/b变体（n=8）有更优的中位PFS（314天 vs 192天，P=0.1743）和中位持续治疗时间（510天 vs 215天，P=0.1080），另一项研究^[70]^也得到相似结果。除ALK融合基因外，RNA-based NGS也可指导MET 14跳突和其他融合基因相关药物的靶向治疗。一项利用DNA-based NGS和RNA-based NGS检测MET 14跳突的I期临床研究^[71]^中接受克唑替尼治疗患者的整体客观缓解率为32%，其中基于RNA检测患者的客观缓解率达到36.4%。此外，针对ROS1、RET、NRG1和NTRK基因融合的前瞻性临床研究也将RNA-based NGS作为指导患者入组用药的诊断标准之一^[3,53,72,73]^。

基于DNA-based NGS检测的驱动基因阴性患者中，RNA-based NGS可以多检出10%-14.2%可用药融合变异^[40,74]^。对于驱动基因阴性患者的一线治疗，目前的中国临床肿瘤学会（Chinese Society of Clinical Oncology, CSCO）指南和NCCN指南通常建议选择免疫单药或者免疫联合治疗^[75,76]^。而现有研究数据显示，NSCLC患者接受一线免疫治疗的PFS和总生存期（overall survival, OS）仍劣于一线融合基因相关的靶向治疗。例如，ALEX研究^[77]^中ALK融合阳性的患者一线接受阿来替尼治疗的中位PFS达34.8个月，中位OS尚未达到。另一项针对ROS1基因融合的研究^[3]^中，患者接受一线克唑替尼治疗的中位PFS为19.3个月，中位OS达51.5个月。而接受一线西米普利单抗联合化疗的患者中位PFS仅8.3个月，中位OS为21.9个月^[78]^。信迪利单抗联合化疗作为一线治疗的研究数据（中位PFS：8.9个月，中位OS：24.2个月）同样不及融合靶向治疗^[79]^。因此，为了降低NSCLC患者错失融合靶向治疗机会的概率，有必要对可用药融合基因进行RNA-based NGS检测。

融合基因参与转录和翻译过程并最终表达出有功能的融合蛋白是导致NSCLC发生发展的原因之一，准确识别出有功能的融合蛋白是患者治疗获益的关键。对于DNA水平检测融合阳性而RNA或蛋白水平检测为阴性的患者，靶向药物治疗可能疗效不佳，应谨慎考虑使用。一项回顾性研究^[49]^通过DNA-based NGS鉴定了14例NSCLC患者的ALK基因复杂融合形式，克唑替尼对ALK基因复杂融合形式的疗效与ALK基因经典融合形式无明显差异，经RNA-based NGS验证后发现由DNA-based NGS鉴定的ALK基因复杂融合形式的样本均为EML4-ALK经典融合转录本。有研究^[43]^发现，由DNA-based NGS检测出ALK基因融合而RNA-based NGS和IHC均未检出的NSCLC患者接受ALK抑制剂治疗后影像学评估为疾病进展，PFS均不超过2个月，提示由DNA-based NGS检测的部分基因融合形式并未发生转录和翻译，即存在非功能性融合，未能给患者带来明显临床获益，而融合基因经RNA-based NGS未检出或许可解释为何ALK抑制剂疗效不佳。也有研究^[6]^发现，由DNA-based NGS检测的部分ALK融合患者经RNA-based NGS或IHC再次验证基因融合变异的患者对克唑替尼的获益持续时间明显长于经RNA-based NGS或IHC未能确认基因融合变异的患者[PFS：11.0个月（95%CI: 8.9-13.1）vs 2.0个月（95%CI: 1.2-2.8）P=0.001]，表明由DNA-based NGS检测且经RNA或蛋白水平确认的融合基因变异部分患者具有更好的靶向治疗获益^[79]^。因此，RNA-based NGS可进一步验证DNA层面检测到的融合是否转录表达，从而降低DNA-based NGS未检出基因融合变异时可能无法从靶向治疗中获益的风险。当RNA-based NGS与IHC、FISH或DNA-based NGS检测出现两种结果不一致时，建议通过第三种检测方法进行验证，以指导临床靶向用药。

**共识四：RNA-based NGS可应用于所有NSCLC人群，同时建议更多关注与融合基因发生频率相关性较高的肺癌患者（如腺癌、女性、不吸烟、肿瘤进展快速等）。【强烈推荐】**

RNA-based NGS可应用于所有NSCLC人群，通过临床病理生理特征有助于识别发生融合基因变异较高的肺癌人群。在一项纳入ALK、ROS1、RET、NTRK基因融合及MET 14跳突检测的研究^[5]^中，在从不吸烟的患者中通过RNA-based NGS检出基因融合的比例显著高于有吸烟史的患者（10/31, 32% vs 7/189, 4%, P<0.01）。多项中国人群的研究^[80⇓-82]^显示，约80%的ALK基因融合发生在腺癌中，且女性的ALK基因融合发生率约为男性的2-3倍。一项大型荟萃分析^[83]^显示，ROS1基因融合更易发生在腺癌（OR=1.55, 95%CI: 1.14-2.11, P<0.05）、女性（OR=1.94, 95%CI: 1.62-2.32, P<0.05）和无吸烟史患者（OR=2.82, 95%CI: 2.24-3.55, P<0.05）中。另一项研究^[84]^显示，277例ROS1基因融合的肿瘤患者中女性占69%，不吸烟患者占75%。多项关于RET基因融合的研究^[72,85⇓⇓⇓-89]^发现，80%以上的RET基因融合发生在腺癌患者中，RET基因融合的女性患者约为男性患者的3倍，从不吸烟患者约为吸烟患者的2倍。一项分析FoundationCORE数据库的研究^[90]^显示，889例NTRK基因融合的肿瘤患者（肺腺癌占比约70%）中女性占比57.5%。另一项研究^[44]^发现NRTK基因融合的不吸烟患者比例占61%。MET 14跳突在肺腺癌中发生率为3%-4%，在肺肉瘤样癌中发生率更高，达到8%-32%^[91,92]^，针对MET 14跳突的临床研究中，女性占比约为60%、不吸烟或从不吸烟的患者占比约为70%^[93]^。针对NRG1融合的研究^[9]^显示，94%为肺腺癌，近60%患者为女性，超过80%患者为不吸烟患者。综上，基于已有的研究数据，ALK/ROS1/RET/NTRK/NRG1等基因融合主要出现在肺腺癌中，MET 14跳突在肺腺癌和肺肉瘤样癌中的发生率均较高，以上靶点均主要集中在女性、不吸烟人群中。因此，对于具有以上临床病理生理学特征的患者，应当更加注重运用RNA-based NGS的检测，尽可能提高靶点检出率。

**共识五：FFPE样本经质控评估合格后可用于RNA-based NGS检测融合基因。【强烈推荐】**

RNA-based NGS检测融合基因优先推荐使用组织学样本，包括经福尔马林固定后制备的FFPE组织样本或新鲜（冰冻）组织标本。FFPE样本是临床实践中最常见的一种样本类型，多年来被广泛用于IHC及分子检测。尽管福尔马林固定和石蜡包埋处理会导致RNA的质量和完整性受损，使FFPE样本提取RNA进行融合基因检测具有一定的技术难度，但随着分子生物学技术的不断进步，越来越多的研究^[94⇓⇓-97]^表明FFPE样本提取RNA进行融合基因检测是可行的，并且与新鲜冰冻样本中检测到的融合具有较高的一致性。对于无法获得组织学标本的情况，细胞学样本如胸腹腔积液等需进行肿瘤细胞量评估或制作成FFPE样本，满足检测需求后可尝试提取RNA进行融合基因检测。新鲜组织样本因未经过长时间的室温暴露或固定包埋处理而RNA降解少、质量高，常作为RNA-based NGS检测首选的标本类型。然而，在临床实践中仅部分医院或科室具有收集、处理和储存这类标本的条件，在常规情况下可及性不高，因此可将新鲜组织标本包埋为FFPE样本后再行检测。FFPE样本中RNA提取和建库步骤需用已经获许上市的商业试剂盒进行。其他样本类型，如血液、脑脊液等在一些情况下被应用于ctDNA液体活检，但目前尚不推荐从液体活检样本中提取RNA进行肿瘤融合基因检测。

FFPE样本的保存时间对RNA提取的完整性、总产量和纯度的影响尚无明确结论。有研究^[98]^表明，收集后5年内的FFPE样本依然可以用于RNA提取和测序分析，但也有研究者^[99]^发现，在收集后的4年内，大约20%的来自FFPE样本经RNA-based NGS检测时可能不适合基于RIN值（RNA integrity number）和DV200的RNA完整性分析。尽管一些研究^[95,99]^报道了从保存3年以上甚至超过10年的FFPE样本中成功提取RNA进行基因检测，但随着FFPE样本保存时间的增加RNA降解的风险增大，使用年限较久的FFPE样本进行RNA分析会面临较大的技术挑战和不确定性，检测的假阴性风险增加，建议尽量使用保存年限较短（3年以内）的标本进行检测。另外，FFPE样本中RNA的质量和完整性还会受到其他多种因素影响，包括离体样本固定是否及时、固定液类型、保存条件和样本厚度等因素^[100]^。因此，临床需要综合评定并且有必要对RNA的总量、降解情况等采取严谨的质量控制措施。组织学标本需经质控合格后用于融合基因检测，以获得更可靠和准确的结果。

**共识六：RNA-based NGS检测融合基因应充分评估肿瘤细胞含量、RNA的完整性、文库产量和纯度等质控信息，需在有资质的医疗机构出具RNA-based NGS检测报告。【强烈推荐】**

已有的研究^[101]^表明：肿瘤细胞含量会影响NGS检测的灵敏度，用于NGS检测的样本肿瘤细胞含量应不低于20%，肿瘤细胞含量过低时可尝试进一步富集后再用于检测^[27,102,103]^。RNA的完整性、文库产量和纯度直接关系到检测结果的准确性，测序前需对RNA完整性、文库产量和纯度进行评估。评估RNA完整性一般有两个常用的指标：RIN值和DV200值。RIN值是通过电泳分析RNA样本中18S和28S rRNA的降解程度而计算得出。RIN值的范围从0到10，RIN值越高代表RNA完整性越高；DV200表示RNA样本中长度大于200个核苷酸的RNA分子占总RNA量的百分比，DV200值越高表明RNA样本中高质量RNA越多，故更适合进行后续实验。RIN值评估更适合样本质量较高的新鲜冰冻样本，一般在基因表达定量的研究中更常使用。对于存在降解的RNA样本RIN值敏感性相对较低，有研究^[104,105]^表明DV200比RIN值更适合FFPE样本的RNA质量评估，DV200与文库产量之间具有更好的相关性。尽管不同的研究中DV200的阈值设置不完全相同，较多研究^[99,106,107]^中使用DV200≥30%作为阈值，样本DV200≥30%方可用于后续进一步的检测。RNA纯度是通过分光光度计测定260/280 nm和260/230 nm吸光度（absorbance, A），核酸的吸光度峰值在260 nm处，蛋白质的吸光度峰值在280 nm处，盐、有机溶剂等物质通常在230 nm处存在吸光度峰值。A260/A280值在2.0附近时，RNA纯度符合标准。A260/A230值通常在2.0到2.2的范围内^[108]^。

需要指出的是，RNA-based NGS检测过程中应有严格的全流程质控，对送检标本病理质量、RNA质量、文库质量、测序深度、下机数据质量和数据分析等各环节设置质控点，并且在报告中体现上述质控信息。建议在医院病理科或拥有美国病理学家学会（College of American Pathologists, CAP）、美国联邦医疗保险和医疗救助服务中心颁发的CLIA（Clinical Laboratory Improvement Amendments）和中国合格评定国家认可委员会（China National Accreditation Service for Conformity Assessment, CNAS）等资质认证的独立实验室开展RNA-based NGS的融合基因检测，以确保检测结果的准确性。

本文结合专家观点及相关研究结果，主要对RNA-based NGS检测NSCLC融合基因的应用时机、应用场景、实践可及性和质控等进行了共识推荐。尽管如此，目前RNA-based NGS检测融合基因仍然存在未解决的问题。例如：缺乏在同一研究中经DNA-based NGS与RNA-based NGS同时检测各个融合基因的头对头研究设计，缺乏FFPE样本储存年限对经RNA-based NGS检测的RNA完整性、纯度、产量和检出率影响的可靠研究证据，缺乏DNA-based NGS与RNA-based NGS检出不一致时融合变异相关靶向治疗的队列研究结果。因此，建议在有条件的情况下开展前瞻性的大样本临床研究。

**Table:** 参与本共识的专家组成员

**本共识编撰指导专家**	
韩宝惠	上海交通大学附属胸科医院
林冬梅	北京大学肿瘤医院
周清华	四川大学华西医院
宋勇	东部战区总医院
周晓燕	复旦大学附属肿瘤医院
周清	广东省人民医院
**执笔专家**	
钟华	上海交通大学附属胸科医院
**参与共识讨论专家** **（按姓氏拼音字母排序）**	
陈瑞	中山大学孙逸仙纪念医院
储天晴	上海交通大学附属胸科医院
董辉	中国人民解放军海军军医大学 第三附属医院
董晓荣	华中科技大学同济医学院 附属协和医院
范松青	中南大学湘雅二医院
郭凌川	苏州大学附属第一医院
郭人花	江苏省人民医院
韩琤波	中国医科大学附属盛京医院
韩昱晨	上海交通大学附属胸科医院
何勇	陆军特色医学中心
胡晓彤	浙江大学医学院附属邵逸夫医院
黄伟哲	汕头大学医学院第二附属医院
蒋莉莉	四川大学华西医院
蒋日成	天津医科大学肿瘤医院
孔令非	河南省人民医院
李剑敏	温州医科大学附属第一医院
李琳	北京医院
李青	常州市第一人民医院
李伟峰	中国人民解放军南部战区总医院
李晓燕	首都医科大学附属北京天坛医院
李勇	南昌大学第一附属医院
李媛	复旦大学附属肿瘤医院
梁晓华	复旦大学附属华山医院
林丽珠	广州中医药大学第一附属医院
刘国龙	广州市第一人民医院
刘军	南通大学附属医院
刘泽兵	上海交通大学医学院附属仁济医院
卢林明	皖南医学院第一附属医院
吕冬青	温州医科大学附属台州医院
吕镗烽	东部战区总医院
马海涛	苏州大学附属第一医院
马铮	重庆市人民医院
彭浩	云南省第一人民医院
任胜祥	同济大学附属上海市肺科医院
师怡	福建医科大学附属肿瘤医院
施云飞	昆明医科大学第一附属医院
王芳	中山大学附属肿瘤医院
王昊飞	南方医科大学南方医院
王佳蕾	复旦大学附属肿瘤医院
王俊	江苏省人民医院
王昆	昆明理工大学附属 安宁市第一人民医院
王琪	大连医科大学附属第二医院
王文祥	湖南省肿瘤医院
王哲海	山东第一医科大学附属肿瘤医院
温永琴	东莞市人民医院
郗彦凤	山西省肿瘤医院
夏国豪	江苏省肿瘤医院
肖海平	广东药科大学附属第一医院
谢彤	广西医科大学附属肿瘤医院
许川	贵州省人民医院
许新华	宜昌市中心人民医院
杨映红	福建医科大学附属协和医院
杨哲	昆明医科大学第一附属医院
尤长宣	南方医科大学南方医院
袁静萍	武汉大学人民医院
岳东升	天津医科大学肿瘤医院
岳君秋	湖北省肿瘤医院
臧远胜	上海长征医院
张呈生	南昌大学第一附属医院
赵军	北京大学肿瘤医院
**致谢** 感谢思路迪科技（上海）有限公司在数据和资料收集过程中提供的帮助。	

**Competing interests**

The authors declare that they have no competing interests.
